# Anti-Inflammatory Activity of Olive Oil Polyphenols—The Role of Oleacein and Its Metabolites

**DOI:** 10.3390/biomedicines10112990

**Published:** 2022-11-21

**Authors:** Vânia Costa, Marlene Costa, Romeu António Videira, Paula Branquinho Andrade, Fátima Paiva-Martins

**Affiliations:** 1 1REQUIMTE/LAQV, Department of Chemistry and Biochemistry, Faculty of Science, University of Porto, Rua do Campo Alegre 687, 4169-007 Porto, Portugal; 2 REQUIMTE/LAQV, Laboratory of Pharmacognosy, Department of Chemistry, Faculty of Pharmacy, University of Porto, Rua de Jorge Viterbo Ferreira nº 228, 4050-313 Porto, Portugal

**Keywords:** oleacein, hydroxytyrosol, metabolites, macrophage, inflammation, COX-1, COX-2, PLA_2_, 5-LOX

## Abstract

The anti-inflammatory potential of oleacein, the main polyphenolic compound found in olive oil, and its main metabolites were characterized by their effects on RAW 264.7 macrophages challenged with lipopolysaccharide (LPS), and by their ability to inhibit enzymes of the arachidonic acid metabolism with a key role in the synthesis of pro-inflammatory lipid mediators. Oleacein at 12.5 µM significantly decreased the amount of L-citrulline and ^●^NO generated by LPS-stimulated macrophages. Hydroxytyrosol, hydroxytyrosol acetate and hydroxytyrosol acetate sulfate were also able to reduce the cellular amount of ^●^NO, although to a lesser extent. In contrast, hydroxytyrosol glucuronide and sulfate did not show detectable effects. Oleacein was also able to inhibit the coupled PLA_2_ + 5-LOX enzyme system (IC_50_ = 16.11 µM), as well as the 5-LOX enzyme (IC_50_ = 45.02 µM). Although with lower activity, both hydroxytyrosol and hydroxytyrosol acetate were also capable of inhibiting these enzymes at a concentration of 100 µM. None of the other tested metabolites showed a capacity to inhibit these enzymes. In contrast, all compounds, including glucuronides and sulfate metabolites, showed a remarkable capacity to inhibit both cyclooxygenase isoforms, COX-1 and COX-2, with IC_50_ values lower than 3 µM. Therefore, oleacein and its metabolites have the ability to modulate ^●^NO- and arachidonic acid-dependent inflammatory cascades, contributing to the anti-inflammatory activity associated with olive oil polyphenols.

## 1. Introduction

Epidemiological data show that adherence to the Mediterranean diet reduces the incidence of chronic human diseases caused by inflammation and cellular oxidation, such as cardiovascular and neurodegenerative diseases [1,2,3]. These health effects have been partially associated with the high concentration of phenols in extra virgin olive oil (EVOO), the most important fat in this diet [1,2,3,4]. The phenolic composition of EVOO is quite complex and includes phenolic alcohols, hydroxytyrosol and tyrosol, and their esters secoiridoids, oleacein and oleocanthal [5,6]. Oleacein is usually the main antioxidant polyphenolic compound found in EVOO and is believed to be responsible in part for the anti-inflammatory activity [7]. This compound is also present in olive leaves and can be obtained in high yields by simple extraction procedures, which makes oleacein a good candidate for the development of new anti-inflammatory drugs [8]. Although the commercially available anti-inflammatory agents allow the efficient control of acute inflammatory responses, their long-term use in the management of chronic inflammatory states is associated with adverse side effects [9]. To overcome these drawbacks, the search for safer inflammation-mitigating agents derived from dietary natural sources is considered a valuable approach [10].

**Figure 1:**
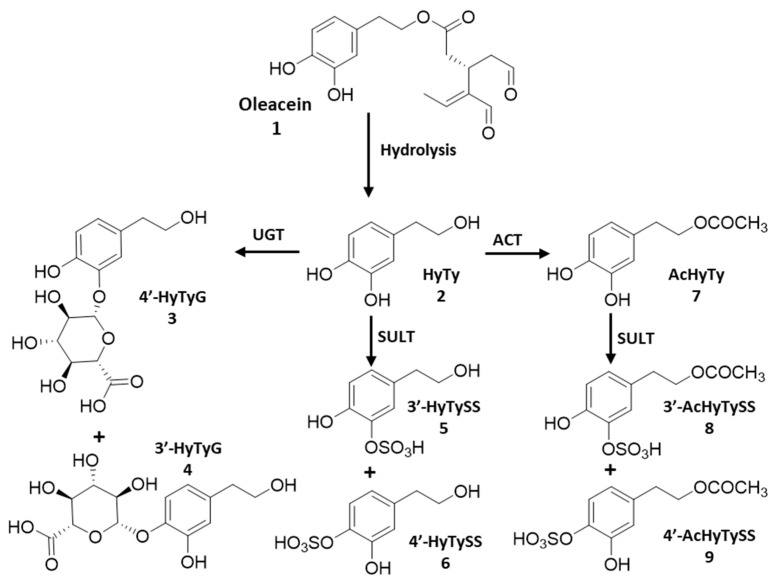
Olive oil polyphenol metabolites identified in bioavailability studies after oleacein consumption. Arrows may represent a direct or an indirect metabolic pathway. ACT—O-acetyltransferase; SULT—sulfotransferase; UGT—glucuronosyltransferase.

The bioactivity of dietary phenolic compounds should be evaluated taking into account their bioavailability once ingested [11]. Secoiridoids are usually subjected to hydrolysis under gastric conditions, with a significant increase in the free hydroxytyrosol and tyrosol, which can be absorbed in the small intestine. Both hydroxytyrosol and tyrosol undergo extensive metabolism in the organism, the acetyl, the O-methyl, the glucuronide and the sulfate conjugates being the main metabolites found in biological fluids, as displayed in Figure 1 [12,13,14].

The aim of this work is to disclose the role of oleacein and its main metabolites on the anti-inflammatory activity assigned to olive oil polyphenol fractions, investigating their ability to modulate the phenotype changes in macrophages induced by LPS and to inhibit enzymes involved in arachidonic acid (AA) metabolism with a key role in the synthesis of pro-inflammatory lipid mediators. Thus, the anti-inflammatory properties of oleacein (**1**), hydroxytyrosol (HyTy, **2**), hydroxytyrosol glucuronides (HyTyG, **3** and 4), hydroxytyrosol sulfate (HyTySS, **5** and **6**), hydroxytyrosol acetate (AcHyTy, **7**) and hydroxytyrosol acetate sulfates (AcHyTySS, **8** and **9**) were assessed by measuring their ability: (i) to modulate the production of ^●^NO and L-citrulline by lipopolysaccharide (LPS)-stimulated murine macrophage-like cells (RAW 264.7); and (ii) to inhibit phospholipase A_2_ (PLA_2_), 5-lipooxygenase (5-LOX) and cyclooxygenases 1 and 2 (COX-1 and COX-2), key enzymes of the arachidonic acid (AA) cascade.

## 2. Materials and Methods

### 2.1. Reagents and Standards

LPS from *Salmonella enterica*, 5-LOX from *Glycine max* (soybean), PLA_2_ from *Apis mellifera* bee venom, sulfanilamide, 3-(4,5-dimethylthiazol-2-yl)-2,5-diphenyltetrazolium bromide (MTT), N-(naphtha-1-yl)ethylenediamine dihydrochloride, trypan blue, propan-2-ol, diacetyl monoxime, dimethyl sulfoxide (DMSO), antipyrine E, quercetin, HEPES, citric acid and sulfuric acid were from Sigma-Aldrich (St. Louis, MO, USA). Phosphoric acid, acetic acid, sodium dihydrogen phosphate dihydrate and L-arginine were obtained from Merck (Darmstadt, Germany). Dulbecco’s Modified Eagle Medium with GlutaMAX^TM^ supplement (DMEM + GlutaMAX), fetal bovine serum (FBS), Hank’s balanced salt solution (HBSS) and Pen Strep solution (penicillin 5000 units/mL and streptomycin 5000 µg/mL) were acquired from Gibco Invitrogen (Grand Island, NE, USA). Sodium chloride and Tris hydrochloride (Tris-HCl) were purchased from Honeywell (Charlotte, NC, USA) and VWR (Radnor, PA, USA), respectively. 1,2-dilauroyl-sn-glycero-3-phosphocholine (DL-PC) was acquired from Larodan (Solna, Sweden). The COX Fluorescent Inhibitor Screening Assay Kit was obtained from Cayman Chemical (Ann Arbor, MI, USA).

### 2.2. Phenolic Compounds and Metabolites

Oleacein and oleocanthal were isolated from olive leaves. Leaves of *Olea europaea* were collected in Oporto, Portugal, in October 2020. Oleacein was isolated, according to the procedure of Paiva-Martins and Gordon [8]. Oleocanthal was isolated following the oleacein isolation procedure, with additional purification of this compound present in the phenolic extract by preparative thin-layer chromatography with a dichloromethane/methanol (5:1, v/v) mixture and ethyl acetate/hexane (1:1, v/v) mixture as eluents. HyTy and AcHyTy were commercially obtained from Seprox Biotech (Madrid, Spain). Monosulfate metabolites, including AcHyTySS and HyTySS, were obtained by synthesis according to the procedure of Gomes et al. [15]. HyTyG were synthesized from HyTy according to Paiva-Martins et al. [16]. In all cases, a mixture of the 3′ and 4′ monoconjugate metabolite isomers was obtained in a proportion of 1:0.8 and were evaluated as a mixture. Compounds were stored at −20 °C and protected from light until use.

### 2.3. RAW 264.7 Macrophages

#### 2.3.1. Cell Culture

Murine macrophage cell line RAW 264.7, from the American Type Culture Collection (LGC Standards S.L.U., Barcelona, Spain), was cultured in flasks using DMEM + GlutaMAX medium supplemented with 10% FBS and 1% Pen Strep solution and maintained at 37 °C in a humidified atmosphere of 5% CO_2_.

#### 2.3.2. MTT Cell Viability Assay

Cell viability was evaluated in 96-well plates at a density of 35,000 cells/well by the MTT reduction assay, as previously described [17]. The compounds were tested in the concentration range of 0–100 µM, and the effects on cell viability of non-activated RAW 264.7 were assessed after 24 h of exposure. After removing the medium, the MTT (0.5 mg/mL) was added to each well and cells were incubated for 90 min at 37 °C. The resulting formazan crystals in each well were then dissolved in 200 µL of DMSO:propan-2-ol (3:1, v/v) and finally quantified spectrophotometrically at 560 nm in a microplate reader (Thermo Scientific Multiskan Go; Waltham, MA, USA). Results were expressed as % of control and correspond to the mean ± standard error of mean (SEM) of four independent assays, each one performed in triplicate.

#### 2.3.3. Determination of ^●^NO Levels in Cell Culture Medium

The effect of compounds on ^●^NO levels in culture medium of LPS-activated macrophages was determined by measuring the nitrites through the Griess reaction, as previously described [18]. Briefly, RAW 264.7 cells were seeded in 96-well plates at a density of 35,000 cells/well. After 24 h, cells were pre-treated with non-toxic concentrations of compounds or media (control) for 1 h and then co-incubated with LPS solution (0.3 µg/mL) for an additional 23 h. Afterwards, ^●^NO levels in cell culture medium were determined by mixing 75 µL of cell culture medium with an equal volume of Griess reagent and quantifying spectrophotometrically at 560 nm in a microplate reader (Thermo Scientific Multiskan Go; Waltham, MA, USA). The results were expressed as % of ^●^NO production by cells exposed to LPS and correspond to the mean ± SEM of four independent assays, each performed in triplicate. Quercetin was used as the positive control.

#### 2.3.4. Determination of L-Citrulline and ^●^NO Levels in Extracellular Medium

L-citrulline was quantified by a colorimetric method that takes advantage of the ureido group-specific reaction with diacetyl monoxime/antipyrine in the presence of sulfuric acid, using cell assay conditions that avoid the interferences promoted by the serum proteins and pH indicator present in cell culture medium. Cells were cultivated for 24 h in 48-well plates at a density of 70,000 cells/well and pre-treated with the compounds that have demonstrated effects on ^●^NO levels (i.e., oleacein, HyTy, AcHyTySS and AcHyTy) for 1 h and then co-incubated with LPS solution (0.3 µg/mL) for an additional 23 h. Afterward, the culture medium was removed, cells were washed three times with HBSS before being incubated with HBSS supplemented with 200 µM of L-arginine (300 µL/well) for an additional 3 h. Then, the extracellular medium of each well was collected and used to assess the ^●^NO and L-citrulline released by cells. 75 µL were used to quantify the ^●^NO levels by using Griess reagent, as described above. For L-citrulline quantification, 250 µL extracellular medium were mixed with 1250 µL of chromogenic reactional solution (40% (v/v) of 79 mM diacetyl monoxime in 83 mM of acetic acid, 18% (v/v) of 47.8 mM antipyrine E in H_2_O and 42% (v/v) of 7.5 M sulfuric acid). The resulting solutions were incubated at 96 °C for 25 min and, after cooling down to room temperature, the absorbance was read at 450 nm in a microplate reader (Thermo Scientific Multiskan Go; Waltham, MA, USA). The results were expressed as % of L-citrulline in cells exposed to LPS and correspond to the mean ± SEM of four independent assays, each one performed in triplicate. Quercetin was used as the positive control.

### 2.4. Arachidonic Acid Cascade Enzymes

#### 2.4.1. PLA_2_ Inhibition Assay

PLA_2_ activity was determined based on an enzymatic assay, which uses DL-PC as the substrate for this enzyme and 5-LOX as the coupling enzyme, as previously described, with some modifitions [18]. PLA_2_ and 5-LOX were used in a concentration of 0.3 µg/mL and 0.4 µL/mL in each well, respectively, dissolved in 10 mM Tris-HCl buffer (pH 7.5). 20 µL of each enzyme were dissolved in 100 µL of the same buffer, with oleacein (3.125–100 µM), AcHyTy (12.5–100 µM), HyTy (100 µM), AcHyTySS (100 µM), HyTyG (100 µM) or HyTySS (100 µM), on a 96-well-plate. The reaction started by adding the PLA_2_ substrate, in a concentration of 600 µM dissolved in 50 mM NaCl buffer, 10 mM HEPES and 2 mM citric acid (pH 6.9). The enzyme activity was followed spectrophotometrically by measuring the increase in absorbance, at 234 nm and 37 °C for 10 min. The results were expressed as % of PLA_2_ + 5-LOX system inhibition and correspond to the mean ± SEM of three independent assays.

#### 2.4.2. 5-LOX Inhibition Assay

5-LOX activity was evaluated by an enzymatic assay, following the oxidation of linoleic acid to its conjugated diene, as previously reported [18]. Compounds that revealed some inhibition of the PLA_2_/5-LOX coupled system was then used in this assay, namely oleacein (3.125–100 µM), AcHyTy (12.5–150 µM) and HyTy (100 µM). Compounds were tested in a mixture containing 20 µL of 5-LOX from Glycine max (100 U/mol) in phosphate buffer (100 mM, pH 9), on a 96 well-plate. After 5 min, at room temperature, the reaction started by adding the substrate, 20 µL of linoleic acid (0.84 mM in ethanol). The oxidation was monitored spectrophotometrically, for 3 min at 234 nm (Thermo Scientific Multiskan Go; Waltham, MA, USA). The results were expressed as % of 5-LOX inhibition and correspond to the mean ± SEM of three independent assays, each performed in duplicate. Quercetin was used as the positive control.

#### 2.4.3. COX-1 and COX-2 Inhibition Assay

COX assays were performed using the COX fluorescent inhibitor assay kit with some modifications. This assay was based on the reaction between prostaglandin G_2_ (PGG_2_), product of COX activity, and 10-acetyl-3,7-dihydroxyphenoxazine (ADHP), producing a highly fluorescent compound, resorufin. Briefly, 60 µL of Tris-HCl buffer (100 mM, pH 8.0), 5 µL of hemin, 5 µL of COX (COX-1 or COX-2) and 5 µL of increasing concentrations of each compound (6.25–100 µM) were added to each well in a black 96-well plate. After 5 min, at room temperature, the reaction started by adding 5 µL of ADHP and 20 µL of substrate (AA 0.5 mM with KOH 2.5 mM) in each well. After 3 min, the fluorescence was measured at 590 nm, setting the excitation wavelength at 535 nm, using a multiplate reader Synergy H1 Biotek. The results were expressed as % of COX inhibition and correspond to the mean ± SEM of four independent assays, each performed in duplicate. SC-560 was used as the positive control for COX-1 and Dup-697 was used as the positive control for COX-2 assay.

### 2.5. Statistical Analysis

Statistical analysis was performed using GraphPad Prism 9.0 software. For the cell assays, one-way analysis of variance (ANOVA) with Dunnet’s test was used to compare significance between samples exposed to the compounds and controls. In all cases, *p* values lower than 0.05 were considered statistically significant. To compare the results of the compounds, the concentration at which a substance exerts half of its maximal inhibitory effect (IC_50_) was calculated.

## 3. Results

### 3.1. Effects on RAW 264.7 Macrophages

**Figure 2:**
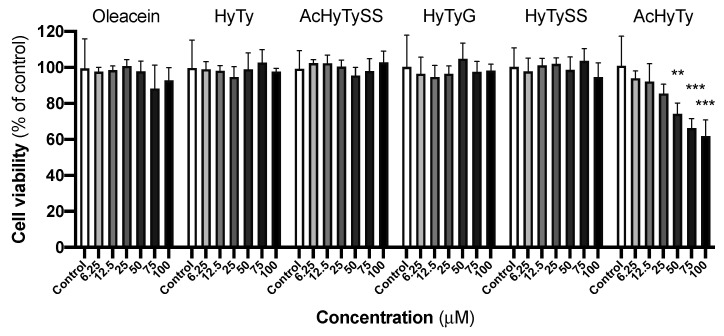
Effect of increasing concentrations of oleacein, HyTy, AcHyTySS, AcHyTy, HyTyG and HyTySS on cell viability of non-activated RAW 264.7 cells, after 24 h of exposure, assessed by MTT reduction. Results are expressed as the mean ± SEM of four independent assays, each performed in triplicate. ** *p* < 0.01; *** *p* < 0.001 compared to the respective control.

As a first approach, non-activated RAW 264.7 macrophages were exposed to increasing concentrations of oleacein, HyTy, AcHyTySS, AcHyTy, HyTyG or HyTySS for 24 h to assess the effects on cell viability by MTT reduction assay (Figure 2). In the range of tested concentrations (0 to 100 µM), oleacein, HyTy, AcHyTySS, HyTyG and HyTySS do not alter the viability of the macrophages, suggesting no cytotoxicity. On the other hand, AcHyTy exhibits cell toxicity for concentrations equal to or higher than 50 µM, as detected by a significant decrease (*p* < 0.01) in cell viability. Regarding the oleacein metabolism represented in Figure 1, the toxicological screening data indicate that only enzymatic conversion of HyTy to the acetate derivative leads to an increase in the cytotoxicity, which is nullified by the subsequent sulfation reaction catalyzed by sulfotransferase.

**Figure 3:**
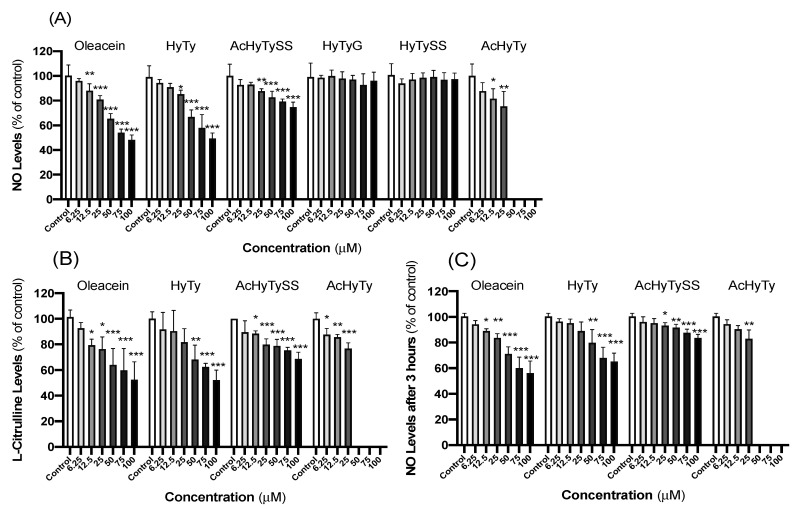
Effects of increasing the concentration of oleacein, HyTy, AcHyTySS, AcHyTy, HyTyG and HyTySS on the production of ^●^NO (**A**), L-Citrulline (**B**) and ^●^NO after 3 h (**C**) by LPS-stimulated RAW 264.7 macrophages. Results are expressed as the mean ± SEM of four independent assays, each performed in triplicate. * *p* < 0.05; ** *p* < 0.01 and *** *p* < 0.001 compared to control. Quercetin was used as the positive control.

Considering the key role of macrophages with a pro-inflammatory activated M1 phenotype on the inflammatory process, the next step was to investigate if oleacein and oleacein metabolites have the competence to modulate, at non-toxic concentrations, the ^∙^NO and L-citrulline generation by LPS-stimulated RAW 264.7 macrophages (Figure 3). Thus, ^●^NO levels released by cells pre-treated for 1 h with the compounds followed by 23 h co-treatment with LPS for culture medium was measured by Griess reagent, and the obtained data are shown in Figure 3A. Oleacein and AcHyTy promote a significant concentration-dependent reduction in ^●^NO levels at concentrations equal to or higher than 12.5 µM. HyTy and AcHyTySS also exhibit the capacity to promote a significant decrease in the ^●^NO levels in cell culture medium; however, the effects only reach statistical significance for concentrations equal to and greater than 25 µM (~20%, *p* < 0.05). In contrast, HyTyG and HyTySS do not affect the ^●^NO generation by LPS-stimulated macrophages, suggesting that the glucuronidation and sulfation reactions catalyzed by phase II enzymes represent an effective mechanism to promote the HyTy inactivation by its conversion in products easily excreted.

The presence of serum proteins and pH indicator in cell culture medium hampers L-citrulline quantification by colorimetric methods. Thus, to overcome these constraints and to confirm if the reduction on ^●^NO levels in the culture medium of LPS-stimulated macrophages detected in the presence of oleacein, HyTy, AcHyTySS and AcHyTy results from cellular effects and not from the ability of the compounds to scavenge nitric oxide, a new set of experiments was performed. To allow the determination of both ^●^NO and L-citrulline levels, a new step was added to the previous experimental design. Cells pre-treated with compounds for 1 h and stimulated for 23 h with LPS were washed and incubated in HBSS supplemented with 200 µM of L-arginine (iNOS substrate) for 3 h. In these assay conditions, the cell metabolism is supported by glucose and the activity of the iNOS enzyme by L-arginine. The levels of L-citrulline and ^●^NO released by cells, during 3 h, for the colorless extracellular medium were quantified, and the results are shown in Figure 3B and Figure 3C, respectively. The data in Figure 3B,C show that oleacein, HyTy, AcHyTy and AcHyTySS decrease the ^●^NO and L-citrulline released by activated macrophages for extracellular medium almost to the same extent, confirming their anti-inflammatory properties towards macrophages. For all compounds, these anti-inflammatory effects are concentration-dependent, but more pronounced for oleacein and HyTy. For example, at the highest tested concentration (100 µM), both oleacein and HyTy decrease by about 50% (*p* < 0.001) the cellular production of ^●^NO and L-citrulline, while AcHyTySS promotes a decrease of about 30% (*p* < 0.001) and AcHyTy of 20% (*p* < 0.01). 

### 3.2. Effects on Arachidonic Acid Cascade

Our strategy to screen the ability of oleacein and oleacein metabolites to modulate the arachidonic acid cascade involved, initially, the assessment of the effects of compounds (at a concentration of 100 µM) on the activity of the PLA_2_ + 5-LOX coupled system and of the 5-LOX pure enzyme. When the inhibitory effects on these enzymatic systems are higher than 20%, additional concentrations of the compound were assessed in order to determine the IC_50_ value. In a second step, the effect of increasing the concentration of oleacein and oleacein metabolites on the activity of COX-1 and COX-2 enzymes was assessed to determine the IC_50_ values for each pair of enzyme/compounds.

#### 3.2.1. Effects on PLA_2_ + 5-LOX Coupled System and on 5-LOX Pure Enzyme

**Figure 4:**
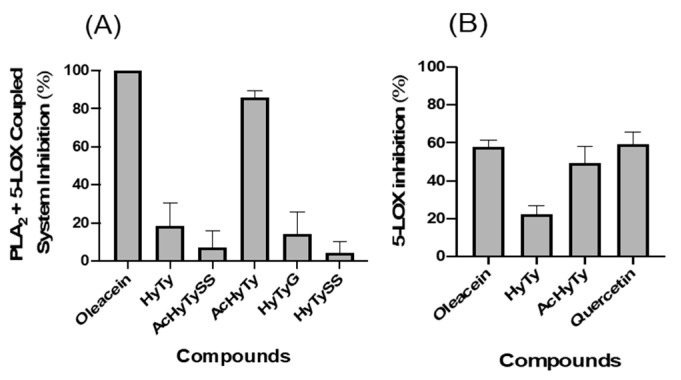
Inhibitory activity of the oleacein, HyTy, AcHyTySS, AcHyTy, HyTyG or HyTySS, at a concentration 100 µM, on the PLA_2_ + 5-LOX coupled system (**A**) and on the 5-LOX enzyme (**B**). Results are expressed as the mean ± SEM of four independent assays, each one performed in duplicate. Quercetin was used as the positive control for the 5-LOX assay.

Figure 4A shows the ability of oleacein, HyTy, AcHyTySS, AcHyTy, HyTyG and HyTySS to inhibit, at a concentration of 100 µM, the PLA_2_ + 5-LOX coupled system. Oleacein and AcHyTy have a high competence to inhibit the PLA_2_ + 5-LOX coupled system, 100% ± 0% and 86.13% ± 3.33%, respectively. HyTy exhibits some inhibitory activity (18.59% ± 11.75%), while the sulfate and glucuronide metabolites (AcHyTySS, HyTyG and HyTySS) did not show significant inhibitory effects on this enzymatic coupled system.

The compounds that showed significant inhibitory effects on the PLA_2_ + 5-LOX coupled system were tested on the pure 5-LOX enzyme, and the obtained results are displayed in Figure 4B. Oleacein and AcHyTy promote an inhibition of the 5-LOX enzyme of 58.13% ± 3.32% and 49% ± 8.38%, respectively, while HyTy show an inhibitory capacity that is 22.19% ± 4.55%. Moreover, the inhibitory activity of the oleacein and AcHyTy is similar to that exhibited by quercetin (59.46% ± 6.08%, at 100 µM), used as the positive control and tested in the same assay conditions. The set of results in Figure 4 indicates that oleacein and AcHyTy are able to inhibit both 5-LOX and PLA_2_ enzymes, while the limited inhibitory effects promoted by HyTy on the PLA_2_ + 5-LOX coupled system results from its direct effects on the 5-LOX enzyme.

**Figure 5:**
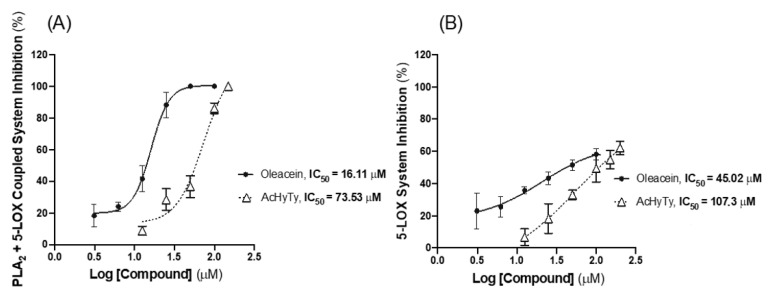
Inhibitory activity of oleacein and AcHyTy on the PLA2 + 5-LOX coupled system (**A**) and 5-LOX pure enzyme (**B**). Results are expressed as the mean ± SEM of four independent assays, each performed in duplicate.

The effects of increasing concentrations of oleacein and AcHyTy on the activity of the PLA_2_ + 5-LOX coupled system and on the pure 5-LOX enzyme are shown in Figure 5A and Figure 5B, respectively. IC_50_ values of 16.11 µM for oleacein and 73.53 µM for AcHyTy were found for the PLA_2_ + 5-LOX coupled system. Regarding the inhibitory effects of the compounds on the 5-LOX, the IC_50_ value determined for oleacein (IC_50_ = 45.02 µM) is about two times lower than the one of AcHyTy (IC_50_ = 107.28 µM), indicating that oleacein is the more effective inhibitor of the enzyme.

#### 3.2.2. Effects on COX-1 and COX-2 Inhibition

**Figure 6:**
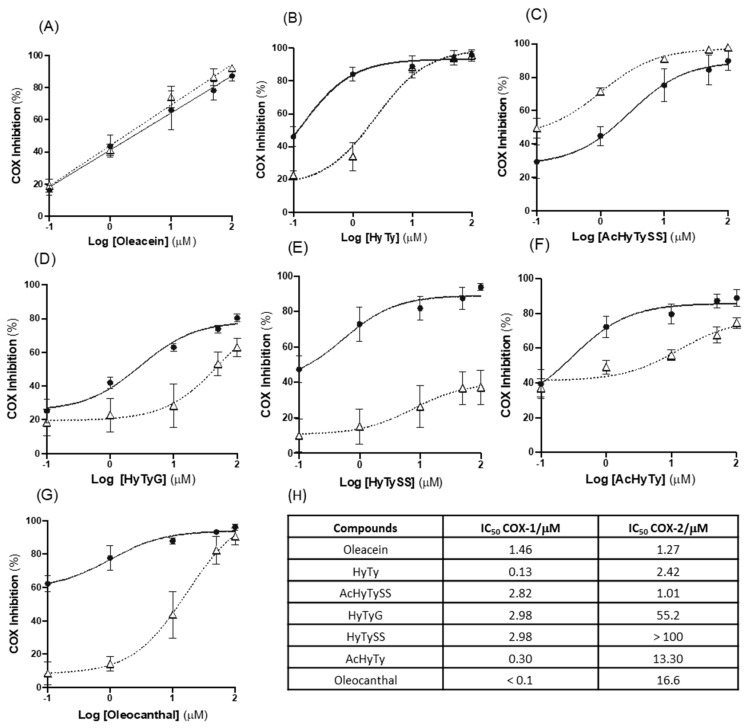
Inhibitory activity of oleacein (**A**), HyTy (**B**), AcHyTySS (**C**), HyTyG (**D**), HyTySS (**E**), AcHyTy (**F**) and Oleocanthal (**G**) on COX-1 (solid lines) and COX-2 (dotted lines) enzymes. Results are expressed as the mean ± SEM of three independent assays, each performed in duplicate. IC_50_ values (µM) of COX-1 and COX-2 enzymes for studied compounds (**H**). SC-560 was used as the positive control for COX-1 and Dup-697 was used as the positive control for the COX-2 inhibition test.

The compounds were tested for their ability to inhibit the COX-1 and COX-2 enzymes’ activity (Figure 6) and compared to that of oleocanthal. Oleocanthal, another secoiridoid found in olive oil, has been described, in previous reports, to have an important inhibitory activity on these cyclooxygenase isoforms, comparable to that of the anti-inflammatory drug ibuprofen [19]. In general, the tested compounds are strong inhibitors of COX-1 enzymes with IC_50_ values lower than 3 µM. Oleocanthal exhibits the best inhibitory activity (IC_50_ < 0.1 µM), followed by HyTy (IC_50_ = 0.13 µM).

In contrast, COX-2 inhibition is more dependent on the compound structure. Thus, AcHyTySS (IC_50_ = 1.01 µM) and oleacein (IC_50_ = 1.27 µM) are very effective COX-2 inhibitors, and HyTySS (IC50 > 100 µM) is the compound with lower inhibitory activity. It is important to highlight that, although the inhibitory capacity of oleacein COX-1 is significantly lower than that of oleocanthal (IC_50_ of 1.46 µM versus IC_50_ < 0.1 µM), it exhibits a COX-2 inhibitory activity that is one order of magnitude greater than that promoted by oleocanthal (IC_50_ = 1.27 µM versus IC_50_ = 16.6 µM). When compared to HyTy, both hydroxytyrosol conjugates, HyTyG and HyTySS, exhibit a decrease in the COX-2 inhibition capacity (IC_50_ of 2.4 µM versus 55.2 and 100 µM, respectively). The data in Figure 6 also show that the conjugation of hydroxytyrosol acetate with the sulfate group produces compounds, AcHyTySS, with higher selectivity for COX-2, by decreasing the inhibitory activity on COX-1 and increasing it on COX-2; thereby, the mixture of AcHyTySS isomers is the best COX-2 inhibitor.

## 4. Discussion

### 4.1. Effects on RAW 264.7 Macrophages

Macrophages are cells with a high plasticity that play a key role in all phases of the inflammatory process. In response to specific external stimuli, macrophages change their physiology from the non-polarized M0 state to a pro-inflammatory activated M1 phenotype that produces and releases different types of signaling molecules (e.g., nitric oxide, protein- and lipid-based inflammatory mediators) required to initiate and/or sustain the inflammatory response. Additionally, activated macrophages in the M1 state can also be deactivated for an anti-inflammatory/pro-resolving (M2) phenotype that undertakes an important role in the resolution of inflammation, as well as in tissue regeneration and repair. Thus, the in vitro approaches to evaluate the anti-inflammatory activity of the compound include the study of its effects on activated macrophages after toxicological screening to find the range of concentrations without toxicity for non-polarized cells [20].

When activated by microbial endotoxins or endogenous inflammatory cytokines, the RAW 264.7 macrophages underwent phenotype remodeling as a consequence of changes in their gene expression pattern, including over expression of the iNOS, which produces high amounts of ^●^NO and L-citrulline using L-arginine and O_2_ as substrates. ^●^NO is a signaling molecule with many physiological functions, but when produced in high amounts via iNOS plays a key role in the pathogenesis of inflammation [21,22]. Thus, compounds with the ability to decrease the ^●^NO levels in the culture medium of LPS-stimulated RAW 264.7 macrophages are considered compounds with an anti-inflammatory capacity.

The cytotoxic screening performed in the first step of the work showed that oleacein, HyTy, AcHyTySS, HyTyG or HyTySS do not affect RAW 264.7 cell viability up to 100 µM, while AcHyTy exhibited cytotoxic effects for concentrations equal to or higher than 50 µM (Figure 2).

The co-exposure of macrophages to LPS and non-toxic concentrations of oleacein, HyTy, AcHyTySS or AcHyTy reduced the ^●^NO levels in a dose-dependent manner, indicating that oleacein, HyTy, as well its acetyl derivative, have significant anti-inflammatory activity at a concentration range of 12.5–25 µM (up to 25%, *p*< 0.05). The reduction in ^●^NO levels was accompanied with the decrease in L-citrulline production by LPS-stimulated macrophages, confirming the effects of these compounds on the modulation of iNOS enzyme activity (Figure 3). These findings are in accordance with those found in the literature for HyTy [23]. In contrast, the conjugated metabolites HyTyG and HyTySS did not exhibit a detectable capacity to modulate the generation of ^●^NO by activated macrophages, and the AcHyTySS showed some significant activity at the concentration of 25 µM (Figure 3). Since oleacein, HyTy and AcHyTy, with free catecholic hydroxyl groups, show better anti-inflammatory activity on LPS-activated macrophages, this feature seems to be important for the observed effect. Nevertheless, a higher liposolubility seems to increase the activity of compounds, probably due to a higher capacity to pass through membranes. In fact, the presence of a sulfate or glucuronic acid group not only hindered the hydroxyl group but also decreased the ability of compounds to cross membranes by passive diffusion due to the attached anionic hydrophilic moiety and specific protein transporters that are usually now required for transportation [24]. The acetylation of HyTySS increases its liposolubility, which may allow some penetration in the cells when compared to HyTySS; however, this effect is not enough to completely counteract the presence of the sulfate group, which seems to be responsible for the lower capacity of AcHyTySS to attenuate the generation of ^●^NO by LPS-stimulated macrophages when compared to AcHyTy. The lower activity observed for this compound at higher concentrations can also be related to a limited hydrolysis of the sulfate group, needed for activity, that may occur inside the cell. The total inactivity shown by HyTyG and HyTySS, which display higher hydrophilia, is probably due to their inability to cross the cell membrane.

### 4.2. Effects on Arachidonic Acid Cascade

Emerging evidence has shown that olive oil phenols’ health-promoting properties are not only due to their free radical scavenging capacity, but also to their ability to target several enzymes involved in the onset of inflammatory processes and to affect the expression of genes involved in the pathogenesis of many diseases and aging [25,26]. The recognition that arachidonic acid derivatives are involved in a number of diseases with an acute or chronic inflammatory background has made the enzymes involved in arachidonic acid metabolism (e.g., cyclooxygenase COX, a lipoxygenase LOX) popular targets to screen the anti-inflammatory activity of compounds [27]. There are distinct enzymes involved in the AA cascade, including PLA_2_, 5-LOX and COXs (COX-1 and COX-2). AA, present in the phospholipids of cell membranes, can be hydrolyzed by the PLA_2_, which is the main precursor of eicosanoids, a group of regulator and pro-inflammatory mediators [5,28]. Under inflammatory conditions, an increased production of prostaglandins from the COX pathway and leukotrienes (LTs) from the 5-LOX pathway occurs [5,9,18].

The established role of the 5-LOX enzyme is the formation of LTs in neutrophils, contributing to (patho)-physiological inflammation. Moreover, the 5-LOX enzyme is also necessary for the biosynthesis of 5-LOX-derived hemiketal (HK) eicosanoids that are believed to be lipid mediators in inflammation [29].

Among the compounds tested, oleacein and AcHyTy demonstrated a high inhibition capacity of the PLA_2_ + 5-LOX coupled system, with IC_50_ values of 16.11 µM and 73.53 µM (Figure 5), respectively. HyTy showed a modest inhibitory capacity (~19% at 100 µM) of this system. Given the higher IC_50_ value found for the inhibition the pure 5-LOX enzyme by oleacein (IC_50_ = 45.02 µM) and AcHyTy (IC_50_ = 107.28 µM) when compared to the corresponding IC_50_ values obtained for the inhibition of the PLA_2_ + 5-LOX coupled system, we can conclude that these compounds are also inhibitors of the PLA_2_ enzyme. In contrast, HyTy inhibited the PLA_2_ + 5-LOX coupled system to the same extent as the pure 5-LOX enzyme (about 20%, at 100 µM), indicating that it is a weak inhibitor of the 5-LOX enzyme without any inhibitory activity on PLA_2_ enzyme. The lower liposolubility of HyTy, when compared with oleacein and AcHyTy, may prevent a better interaction with the hydrophobic catalytic core of these enzymes.

On the other hand, all sulfate and glucuronide metabolites, AcHyTySS, HyTyG and HyTySS, did not show a significant inhibitory activity of the PLA_2_ + 5-LOX coupled system, even at 100 µM, suggesting that the presence of free catechol moiety is important for the compound’s inhibitory activity on this enzymatic system. The 5-LOX catalytic reaction involves an initial hydrogen abstraction from carbon 7 of AA and the addition of molecular oxygen to produce 5S-hydroperoxyeicosatetraenoic acid (5S-HPETE), followed by a second hydrogen abstraction from position 10 to form LTA_4_ [29]. Phenolic compounds are believed to exert their protective effects through their radical scavenging activity [3], and the conjugation of an aromatic hydroxyl group by a sulfate or a glucuronic acid moiety deeply decreases this capacity. Accordingly, it is understandable that conjugated compounds do not show inhibitory activity. However, the mechanisms by which phenolic compounds modulate 5-LOX activity (and the inflammatory response) may go beyond their radical scavenging activity, and their activity in vivo may be different, as hydrolysis of conjugated metabolites may occur in living tissues. Although the parental phenols are hardly found in the plasma, their presence in inflammatory environments is conceivable via deconjugation, as described for curcumin, quercetin, luteolin, resveratrol and Uro-A [30,31,32,33,34,35,36]. Moreover, after regular oleacein intake by rats, both HyTy and oleacein have been found in free form in high concentrations in several tissues, such as the stomach, small intestine, liver and heart [37].

There are two main distinct isoforms of cyclooxygenase, COX-1 and COX-2, and while the COX-1 isoform is constitutively expressed in most mammalian tissues, since it is involved in their homeostasis, the COX-2 isoform is referred to as an ‘inducible isoform’, which was believed to be undetectable in most normal tissues, and its expression is induced by pro-inflammatory stimuli in macrophage and endothelial cells, among other cells [10,18]. However, previous studies indicate that both isoforms, not only COX-1, are present in many normal human tissues (such as liver, lung, thyroid gland, spleen, adipose tissue, where the expression of COX-2 mRNA has been shown to be equal to or greater than the expression of COX-1), and that both cyclooxygenase isoforms, not only COX-2, are up-regulated in various inflammatory conditions [38].

In general, all tested compounds are effective inhibitors of COX-1, as indicated by the IC_50_ values lower than 3 µM. Oleocanthal, a monohydroxyphenol with a similar structure to oleacein (Figure 1), showed the best inhibitory activity (IC_50_ < 0.01 µM), followed by HyTy (IC_50_ = 0.13 mM), AcHyTy (IC_50_ = 0.30 µM) and oleacein (IC_50_ = 1.46 µM). The conjugation reaction that HyTy and AcHyTy can undergo a decrease 10 to 23 times the inhibitory activity of the resulting compounds, but the IC_50_ values found for these metabolites (IC_50_ values close to 3 µM) were still in the range of values found for many anti-inflammatory drugs. Regarding the COX-1 inhibitory activity promoted by these compounds, we suggest that oleacein and oleacein metabolites have potential for the development of drugs with an antiplatelet aggregation capacity, useful in the treatment and prevention of cardiovascular pathologies.

On the other hand, the ability of these compounds to inhibit the COX-2 enzyme is highly dependent on the compound structure, with oleacein and AcHyTySS showing the best inhibitory activity (IC_50_ values of 1.27 and 1.01 µM, respectively). Moreover, oleacein exhibits an inhibitory activity on COX-2 enzyme 13 times greater than oleocanthal (IC_50_ = 1.27 µM versus IC_50_ = 16.6 µM), despite its inhibitory activity on COX-1 being one order of magnitude lower. The conjugation reactions of HyTy produce compounds, HyTyG or HyTySS, with significantly lower COX-2 inhibitory activity than HyTy (IC_50_ of 2.4 µM versus 55.2 or 100 µM, respectively). In contrast, the conjugation of AcHyTy with the sulfate group originates compounds, AcHyTySS, that exhibit improved COX-2 selectivity and inhibitory activity (IC_50_ = 1.01 µM). In fact, the IC_50_ values (<3 µM) found for AcHyTySS, oleacein and HyTy are lower than the ones reported for several anti-inflammatory drugs such as resveratrol, propenoic acid derivatives and thiophene derivatives [39]. Considering that hydroxytyrosol acetate monosulfates, AcHyTySS, are some of the most important metabolites of HyTy found in vivo, and that hydroxytyrosol metabolites may achieve a plasmatic concentration of 5–10 µM, these metabolites are likely to have a great contribution to the anti-inflammatory effects of regular olive oil consumption [40].

## 5. Conclusions

Non-steroidal anti-inflammatory drugs (NSAIDs), such as ibuprofen, normally lead to gastric mucosal damage due to COX-1 inhibition, since the prostaglandins produced by this enzyme in the gastric epithelium act as cytoprotective agents [39,41]. Therefore, selective COX-2 inhibitors were thought to present lower gastrointestinal toxicity. However, it was found that COX-2 inhibitors are responsible for cardiotoxicity and cardiovascular events because blocking the COX-2 pathway switches the metabolism, exacerbating COX-1 and 5-LOX pathways, lowering PGI2 and increasing TxA2 and LTs production, which increase platelet aggregation, vasoconstriction and inflammatory events [9,39]. In fact, compounds acting on individual molecular targets have been found to produce undesired activities and toxicity, whereas compounds that act on multiple targets concurrently produce a better therapeutic profile [9]. Thus, dual inhibition of LOX/COX has been suggested to be a desirable approach in the development of new drugs for anti-inflammation [9]. Some compounds, such as darbufelone and licofelone, were already designed and clinically used as a dual COX/5-LOX inhibitory drug; however, due to the high toxicity and/or limited efficacy, they were unable to be marketed [10]. As it is known that natural products and their derivates are potentially safer and more active than synthetic compounds, compounds from natural sources, for example olive oil, and their by-products, may be potential inhibitors [10]. Accordingly, oleacein has shown to be a multiple-target drug, able to decrease the production of ^●^NO by LPS-stimulated macrophages and to inhibit key enzymes of the AA cascade, including PLA_2_, 5-LOX, COX-1 and COX-2. Moreover, oleacein metabolites, namely HyTy and AcHyTySS, also exhibit important anti-inflammatory properties detected in both cellular and enzymatic systems; thereby, the in vivo oleacein metabolization extends its anti-inflammatory activity. The present results expand the knowledge on the anti-inflammatory activity associated with olive oil polyphenols and suggests important clues for the development of new anti-inflammatory drugs based on natural compounds.

## References

[1] Carluccio M.A., Calabriso N., Scoditti E., Massaro M., De Caterina R., Preedy V.R., Watson R.R. (2015). Chapter 27—Mediterranean Diet Polyphenols. The Mediterranean Diet.

[2] Boskou D., Boskou D. (2015). 1—Olive Fruit, Table Olives, and Olive Oil Bioactive Constituents. Olive and Olive Oil Bioactive Constituents.

[3] Visioli F., Galli C. (1998). Olive Oil Phenols and Their Potential Effects on Human Health. J. Agric. Food Chem..

[4] Ghanbari R., Anwar F., Alkharfy K.M., Gilani A.H., Saari N. (2012). Valuable nutrients and functional bioactives in different parts of olive (*Olea europaea* L.)—A review. Int. J. Mol. Sci..

[5] Paiva-Martins F., Kiritsakis A., Kiritsakis A., Shahidi F. (2017). Olive fruit and olive oil composition and their functional compounds. Olives and Olive Oil as Functional Foods: Bioactivity, Chemistry and Processing.

[6] Boskou D., Blekas G., Tsimidou M., Boskou D. (2006). 4—Olive Oil Composition. Olive Oil.

[7] Kiritsakis K., Gerasopoulos D., Kiritsakis A., Shahidi F. (2017). Production of Phenol-Enriched Olive Oil. Olives and Olive Oil as Functional Foods: Bioactivity, Chemistry and Processing.

[8] Paiva-Martins F., Gordon M.H. (2001). Isolation and characterization of the antioxidant component 3,4-dihydroxyphenylethyl 4-formyl-3-formylmethyl-4-hexenoate from olive (*Olea europaea*) leaves. J. Agric. Food Chem..

[9] Jaismy Jacob P., Manju S.L., Ethiraj K.R., Elias G. (2018). Safer anti-inflammatory therapy through dual COX-2/5-LOX inhibitors: A structure-based approach. Eur. J. Pharm. Sci..

[10] Nguyen H.T., Vu T.-Y., Chandi V., Polimati H., Tatipamula V.B. (2020). Dual COX and 5-LOX inhibition by clerodane diterpenes from seeds of *Polyalthia longifolia* (Sonn.) Thwaites. Sci. Rep..

[11] Vissers M.N., Zock P.L., Katan M.B. (2004). Bioavailability and antioxidant effects of olive oil phenols in humans: A review. Eur. J. Clin. Nutr..

[12] Corona G., Tzounis X., Assunta DessÌ M., Deiana M., Debnam E.S., Visioli F., Spencer J.P.E. (2006). The fate of olive oil polyphenols in the gastrointestinal tract: Implications of gastric and colonic microflora-dependent biotransformation. Free Radic. Res..

[13] Covas M.-I., Fitó M., Khymenets O., de la Torre R., Preedy V.R., Watson R.R. (2010). Chapter 73—The Bioavailability of Olive Oil Phenolic Compounds. Olives and Olive Oil in Health and Disease Prevention.

[14] Pinto J., Paiva-Martins F., Corona G., Debnam E.S., Jose Oruna-Concha M., Vauzour D., Gordon M.H., Spencer J.P.E. (2011). Absorption and metabolism of olive oil secoiridoids in the small intestine. Br. J. Nutr..

[15] Gomes V.P.M., Torres C., Rodríguez-Borges J.E., Paiva-Martins F. (2015). A Convenient Synthesis of Hydroxytyrosol Monosulfate Metabolites. J. Agric. Food Chem..

[16] Paiva-Martins F., Silva A., Almeida V., Carvalheira M., Serra C., Rodrígues-Borges J.E., Fernandes J., Belo L., Santos-Silva A. (2013). Protective Activity of Hydroxytyrosol Metabolites on Erythrocyte Oxidative-Induced Hemolysis. J. Agric. Food Chem..

[17] Mosmann T. (1983). Rapid colorimetric assay for cellular growth and survival: Application to proliferation and cytotoxicity assays. J. Immunol. Methods.

[18] Pereira R.B., Pereira D.M., Jiménez C., Rodríguez J., Nieto R.M., Videira R.A., Silva O., Andrade P.B., Valentão P. (2019). Anti-Inflammatory Effects of 5α,8α-Epidioxycholest-6-en-3β-ol, a Steroidal Endoperoxide Isolated from Aplysia depilans, Based on Bioguided Fractionation and NMR Analysis. Mar. Drugs.

[19] Beauchamp G.K., Keast R.S., Morel D., Lin J., Pika J., Han Q., Lee C.H., Smith A.B., Breslin P.A. (2005). Phytochemistry: Ibuprofen-like activity in extra-virgin olive oil. Nature.

[20] Veloso C., Videira R.A., Andrade P.B., Cardoso C., Vitorino C. (2022). Topical fixed-dose combinations: Current in vitro methodologies for pre-clinical development. Int. J. Pharm..

[21] Fujiwara N., Kobayashi K. (2005). Macrophages in inflammation. Curr. Drug Targets Inflamm. Allergy.

[22] Andrade C., Ferreres F., Gomes N.G.M., Duangsrisai S., Srisombat N., Vajrodaya S., Pereira D.M., Gil-Izquierdo A., Andrade P.B., Valentão P. (2019). Phenolic Profiling and Biological Potential of Ficus curtipes Corner Leaves and Stem Bark: 5-Lipoxygenase Inhibition and Interference with NO Levels in LPS-Stimulated RAW 264.7 Macrophages. Biomolecules.

[23] Bigagli E., Cinci L., Paccosi S., Parenti A., D’Ambrosio M., Luceri C. (2017). Nutritionally relevant concentrations of resveratrol and hydroxytyrosol mitigate oxidative burst of human granulocytes and monocytes and the production of pro-inflammatory mediators in LPS-stimulated RAW 264.7 macrophages. Int. Immunopharmacol..

[24] Costa M., Sezgin-Bayindir Z., Losada-Barreiro S., Paiva-Martins F., Saso L., Bravo-Díaz C. (2021). Polyphenols as Antioxidants for Extending Food Shelf-Life and in the Prevention of Health Diseases: Encapsulation and Interfacial Phenomena. Biomedicines.

[25] Menendez J.A., Joven J., Aragonès G., Barrajón-Catalán E., Beltrán-Debón R., Borrás-Linares I., Camps J., Corominas-Faja B., Cufí S., Fernández-Arroyo S. (2013). Xenohormetic and anti-aging activity of secoiridoid polyphenols present in extra virgin olive oil: A new family of gerosuppressant agents. Cell Cycle.

[26] Parkinson L., Cicerale S. (2016). The Health Benefiting Mechanisms of Virgin Olive Oil Phenolic Compounds. Molecules.

[27] Wang B., Wu L., Chen J., Dong L., Chen C., Wen Z., Hu J., Fleming I., Wang D.W. (2021). Metabolism pathways of arachidonic acids: Mechanisms and potential therapeutic targets. Signal Transduct. Target. Ther..

[28] Kim J., Lee K.W., Lee H.J., Watson R.R., Preedy V.R., Zibadi S. (2014). Chapter 29—Polyphenols Suppress and Modulate Inflammation: Possible Roles in Health and Disease. Polyphenols in Human Health and Disease.

[29] Giménez-Bastida J.A., González-Sarrías A., Laparra-Llopis J.M., Schneider C., Espín J.C. (2021). Targeting Mammalian 5-Lipoxygenase by Dietary Phenolics as an Anti-Inflammatory Mechanism: A Systematic Review. Int. J. Mol. Sci..

[30] Ávila-Gálvez M., González-Sarrías A., Martínez-Díaz F., Abellán B., Martínez-Torrano A.J., Fernández-López A.J., Giménez-Bastida J.A., Espín J.C. (2021). Disposition of Dietary Polyphenols in Breast Cancer Patients’ Tumors, and Their Associated Anticancer Activity: The Particular Case of Curcumin. Mol. Nutr. Food Res..

[31] Shimoi K., Saka N., Nozawa R.T., Satô M., Amano I., Nakayama T., Kinae N. (2001). Deglucuronidation of a flavonoid, luteolin monoglucuronide, during inflammation. Drug Metab. Dispos. Biol. Fate Chem..

[32] Galindo P., Rodriguez-Gómez I., González-Manzano S., Dueñas M., Jiménez R., Menéndez C., Vargas F., Tamargo J., Santos-Buelga C., Pérez-Vizcaíno F. (2012). Glucuronidated quercetin lowers blood pressure in spontaneously hypertensive rats via deconjugation. PLoS ONE.

[33] Ishisaka A., Kawabata K., Miki S., Shiba Y., Minekawa S., Nishikawa T., Mukai R., Terao J., Kawai Y. (2013). Mitochondrial dysfunction leads to deconjugation of quercetin glucuronides in inflammatory macrophages. PLoS ONE.

[34] Kawai Y., Nishikawa T., Shiba Y., Saito S., Murota K., Shibata N., Kobayashi M., Kanayama M., Uchida K., Terao J. (2008). Macrophage as a target of quercetin glucuronides in human atherosclerotic arteries: Implication in the anti-atherosclerotic mechanism of dietary flavonoids. J. Biol. Chem..

[35] Menendez C., Dueñas M., Galindo P., González-Manzano S., Jimenez R., Moreno L., Zarzuelo M.J., Rodríguez-Gómez I., Duarte J., Santos-Buelga C. (2011). Vascular deconjugation of quercetin glucuronide: The flavonoid paradox revealed?. Mol. Nutr. Food Res..

[36] Ávila-Gálvez M.A., Giménez-Bastida J.A., González-Sarrías A., Espín J.C. (2019). Tissue deconjugation of urolithin A glucuronide to free urolithin A in systemic inflammation. Food Funct..

[37] López-Yerena A., Pérez M., Vallverdú-Queralt A., Miliarakis E., Lamuela-Raventós R.M., Escribano-Ferrer E. (2021). Oleacein Intestinal Permeation and Metabolism in Rats Using an In Situ Perfusion Technique. Pharmaceutics.

[38] Zidar N., Odar K., Glavac D., Jerse M., Zupanc T., Stajer D. (2009). Cyclooxygenase in normal human tissues—Is COX-1 really a constitutive isoform, and COX-2 an inducible isoform?. J. Cell. Mol. Med..

[39] Arora M., Choudhary S., Singh P.K., Sapra B., Silakari O. (2020). Structural investigation on the selective COX-2 inhibitors mediated cardiotoxicity: A review. Life Sci..

[40] Bernini R., Mincione E., Barontini M., Crisante F. (2008). Convenient synthesis of hydroxytyrosol and its lipophilic derivatives from tyrosol or homovanillyl alcohol. J. Agric. Food Chem..

[41] Macedo T., Ferreres F., Pereira D.M., Oliveira A.P., Gomes N.G.M., Gil-Izquierdo Á., Valentão P., Araújo L., Andrade P.B. (2021). Cassia sieberiana DC. leaves modulate LPS-induced inflammatory response in THP-1 cells and inhibit eicosanoid-metabolizing enzymes. J. Ethnopharmacol..

